# *In silico* Prediction of miRNA Interactions With Candidate Atherosclerosis Gene mRNAs

**DOI:** 10.3389/fgene.2020.605054

**Published:** 2020-11-04

**Authors:** Dina Mukushkina, Dana Aisina, Anna Pyrkova, Alma Ryskulova, Siegfried Labeit, Anatoliy Ivashchenko

**Affiliations:** ^1^Department of Biotechnology, Al-Farabi Kazakh National University, Almaty, Kazakhstan; ^2^Department of microbiology, Kazakh Medical University of Continuing Education, Almaty, Kazakhstan; ^3^Medical Faculty Mannheim, University of Heidelberg, Heidelberg, Germany

**Keywords:** gene, atherosclerosis, miRNA, mRNA, association, binding sites cluster, marker

## Abstract

The involvement of genes and miRNAs in the development of atherosclerosis is a challenging problem discussed in recent publications. It is necessary to establish which miRNAs affect the expression of candidate genes. We used known candidate atherosclerosis genes to predict associations. The quantitative characteristics of interactions of miRNAs with mRNA candidate genes were determined using the program, which identifies the localization of miRNA binding sites in mRNA, the free energy interaction of miRNA with mRNA. In mRNAs of *GAS6* and *NFE2L2* candidate genes, binding sites of 21 miRNAs and of 15 miRNAs, respectively, were identified. In *IRS2* mRNA binding sites of 25 miRNAs were located in a cluster of 41 nt. In *ADRB3, CD36, FASLG, FLT1, PLA2G7*, and *PPARGC1A* mRNAs, clusters of miR-466, ID00436.3p-miR, and ID01030.3p-miR BS were identified. The organization of overlapping miRNA binding sites in clusters led to their compaction and caused competition among the miRNAs. The binding of 53 miRNAs to the mRNAs of 14 candidate genes with free energy interactions greater than −130 kJ/mole was determined. The miR-619-5p was fully complementary to *ADAM17* and *CD36* mRNAs, ID01593.5p-miR to *ANGPTL4* mRNA, ID01935.5p-miR to *NFE2L2*, and miR-5096 to *IL18* mRNA. Associations of miRNAs and candidate atherosclerosis genes are proposed for the early diagnosis of this disease.

## Introduction

The diagnosis, prevention and treatment of atherosclerosis are important tasks of modern medicine (Byrne et al., 2014; Churov et al., 2019; Solly et al., 2019; Shoeibi, 2020). In most cases, polygenic diseases, including atherosclerosis, develop when the expression of different combinations of candidate genes changes. As a result of a review of various publications, it seems that the approaches used to search for markers for the diagnosis and treatment of atherosclerosis, including miRNA, have not yet solved the problem of diagnosing and treating atherosclerosis (Chen et al., 2020; Li et al., 2020; Ryu et al., 2020; Sun et al., 2020; Wang W. et al., 2020; You et al., 2020). In human, it is known about 7000 miRNA and more than 20,000 genes, and it is unknown how many miRNA and genes from them participate in the development of atherosclerosis (Byrne et al., 2014; Toba et al., 2014; Lu et al., 2018; Liu et al., 2020; Shi et al., 2020). As a rule, in publications several miRNA and some candidate genes are studied, so the combination of such attempts is large (Wang C. et al., 2020; Wang M. et al., 2020; Zhang et al., 2020). Considering the limitations of existing prediction methods, we can say that the establishment of adequate miRNA markers for diseases is unrealistic using existing approaches in the coming years. This statement is confirmed by the fact that over 20 years of studying miRNA participation in various diseases, diagnostic methods and therapeutic ways of treating diseases with miRNA are not used. Most researches study only the correlation between changes in miRNA concentration and expression of candidate genes, but this approach does not establish specific associations of miRNA and target gene. A number of publications argue that miRNA is the cause of disease without understanding that changes in miRNA concentration can occur through independent expression from intergenic regions or from host gene introns (Madrigal-Matute et al., 2013; Andreou et al., 2015; Feinberg and Moore, 2016; Solly et al., 2019). In all cases, miRNAs appear to modify the expression of target genes and not to cause the disease themselves. It is not taken into account that one miRNA can affect several or even 100s of genes, and one gene may be a target of several miRNAs (Atambayeva et al., 2017; Kondybayeva et al., 2018). In this article, we discuss what should be taken into consideration when reviewing the problem of miRNA interaction with candidate genes and show the need to use a systematic approach in establishing the most probable associations of miRNA and target genes.

It has been found that miRNAs, which are nanoscale regulatory biomolecules, are involved in many biological processes at all stages of the development of atherosclerosis, from early endothelial dysfunction to the erosion and rupture of an unstable atherosclerotic plaque (Cipollone et al., 2011; Madrigal-Matute et al., 2013; Andreou et al., 2015; Maitrias et al., 2015; Feinberg and Moore, 2016; Santovito et al., 2016; Chen et al., 2018; Ren et al., 2018; Wang et al., 2018). In addition, miRNAs are considered novel non-invasive biomarkers of the instability of atherosclerotic plaques and have been associated ischemic disorders (Churov et al., 2019). Their detection in the blood of patients may be a promising direction for the diagnosis of atherosclerosis complications such as ischemic stroke and myocardial infarction (Kanuri et al., 2018; Desjarlais et al., 2019; Moghaddam et al., 2019; Vargas-Alarcon et al., 2019; Velle-Forbord et al., 2019; Wiese et al., 2019; Kondybayeva et al., 2020). When the pathogenic role of a specific miRNA is confirmed, it can be considered a potential therapeutic target (Friedman et al., 2009; Fang et al., 2013; Moghaddam et al., 2019; Kazemi et al., 2020). According to the miRBase database, miRNAs have been found in many human tissues and are able to regulate the expression of more than 60% of all protein-coding genes (Kanuri et al., 2018; Ren et al., 2018; Wiese et al., 2019). Full complementarity between miRNA and mRNA results in degradation (Leidinger et al., 2012; Alagia and Eritja, 2016). However, incomplete complementarity is most often observed, in which case miRNAs suppress translation, generally by binding to the 3′-untranslated regions (3′ UTRs) of mRNAs (Ivashchenko et al., 2014a,c; Alagia and Eritja, 2016; Atambayeva et al., 2017). In addition, miRNAs can bind to other regions of target mRNAs, including the 5′-untranslated regions (5′ UTR) and coding sequence (CDS) (Orom et al., 2008; Forman and Coller, 2010; Ivashchenko et al., 2013; Zhou and Rigoutsos, 2014; Niyazova et al., 2015; Kondybayeva et al., 2018; Yurikova et al., 2019). We used a program that effectively determines the quantitative characteristics of the interaction of miRNA with mRNA and allows us to identify fundamentally new properties of the binding of miRNA to mRNA. The aim of this work was to identify associations between miRNAs and mRNA candidate genes of atherosclerosis for use as markers for the diagnosis of this disease.

## Materials and Methods

The nucleotide sequences of 2565 miRNAs (we name this set as ‘old miRNAs’) were downloaded from the miRBase database^1^ (Release 22.1) and 3707 miRNAs (we name this set as ‘new miRNAs’) obtained from a report by Londin et al. (2015). Due to this work the number of known miRNAs had more than doubled. The nucleotide sequences of genes were obtained from GenBank^2^. A database of 68 candidate genes including the names of the genes and publication sources was compiled, confirming the associations of these genes with atherosclerosis (Supplementary Table S1). A search for the target genes of miRNAs was performed using the MirTarget program (Ivashchenko et al., 2014b). This program determines the following binding characteristics: the start of the miRNA binding site (BS) of mRNA; the locations of miRNA BS (3′ UTR, 5′ UTR, CDS); the interaction free energy (ΔG, kJ/mole); and nucleotide interaction schemes between miRNAs and mRNAs. The ratio of ΔG/ΔGm (%) was determined for each BS, where ΔGm is equal to the free energy binding of miRNA with its full complementary nucleotide sequence. The MirTarget program found hydrogen bonds between adenine (A) and uracil (U), guanine (G) and cytosine (C), G and U, A and C. The distances between A and C were equal 1.04 nanometers, between G and C, and between A and U were equal 1.03 nanometers, between G and U equal to 1.02 nanometers (Leontis et al., 2002). The numbers of hydrogen bonds in the G-C, A-U, G-U, and A-C interactions were found to be 3, 2, 1 and 1, respectively (Kool, 2001; Lemieux and Major, 2002). The MirTarget program determines single miRNA BS in mRNA and miRNA BS which are in clusters (BS arranged with overlapping of nucleotide sequences of the same or several miRNAs) (Aisina et al., 2019). Predicted by the MirTarget program binding sites in over 30 genes were confirmed experimentally (Yurikova et al., 2019).

## Results

The BSs of the miRNAs and mRNAs of the target genes were not uniform along the length of the mRNAs. Both multiple and single BS were identified. BSs could be sequential or overlap with each other. Having overlapping nucleotide sequences in a cluster lead to the compaction of the mRNA sequence that is the target of several miRNAs. When the ΔG and ΔG/ΔGm values of miRNA BS are close to each other, it can be assumed that in the presence of equal miRNA concentrations, the miRNAs with a larger number of BS will be more likely to bind to the mRNAs of target genes. When the miRNA-mRNA interaction strength and the degree of their complementarity are similar, the miRNA has the highest concentration upon binding.

### Characteristics of miRNA Interactions in the 5′ UTRs of the mRNAs of Atherosclerosis Candidate Genes

BSs of miRNAs were identified in the 5′ UTRs of 14 mRNAs. The *GAS6* mRNA contains a large cluster with BS of 21 different miRNAs, half of which have two or three BS, while the rest represent single BS (Table 1). The cluster size is 39 nt, starting at the 11 nt position and ending at position 49 nt. The total BS length in the cluster is 824 nt, where the degree of compaction is 21. This compaction allowed this number of miRNA BS to be located in a 5′ UTR with a length of 153 nt. In an association analysis of seven miRNAs with the *GAS6* mRNA the free energy interaction of the miRNAs with the mRNA is more than −130 kJ/mole, which indicates that these associations are promising markers of atherosclerosis.

**TABLE 1:** Characteristics of miRNAs interaction in the 5′ UTR of mRNA of *GAS6, NFE2L2*, and *SCAP* genes.

miRNA	Start of site, nt	ΔG, kJ/mole	ΔG/ΔGm, %	Length, nt
***GAS6***				
ID00061.3p-miR	17÷23 (3)	−129	94	22
ID00296.3p-miR	17	−144	92	25
ID00457.3p-miR	14÷20 (2)	−123÷−127	91÷94	22
ID00522.5p-miR	17	−125	89	23
ID01041.5p-miR	20	−132	90	24
ID01106.5p-miR	21	−132	89	24
ID01155.3p-miR	17÷23 (3)	−129	94	22
ID01641.3p-miR	17	−134	90	24
ID01702.3p-miR	16÷21 (3)	−136÷−142	86÷91	25
ID01804.3p-miR	11÷20 (3)	−136÷−142	85÷93	25
ID01873.3p-miR	20÷23 (2)	−123÷−125	94÷95	21
ID01879.5p-miR	22	−123	91	22
ID02064.5p-miR	24	−123	94	21
ID02084.3p-miR	22÷25 (2)	−129÷−132	86÷87	24
ID02187.5p-miR	15÷19 (3)	−123	89	23
ID02294.5p-miR	16÷19 (2)	−132÷−136	90÷93	24
ID02538.3p-miR	24	−123	92	22
ID02950.3p-miR	13	−125	89	23
ID03367.5p-miR	17÷23 (2)	−117	93	20
miR-3960	23	−115	92	20
***NFE2L2***				
ID01935.5p-miR	271	−142	100	24
ID00061.3p-miR	444÷450 (3)	−125÷−134	91÷97	22
ID00296.3p-miR	441÷448 (3)	−134÷−140	85÷89	25
ID00457.3p-miR	444	−123	91	22
ID00522.5p-miR	438	−125	89	23
ID01041.5p-miR	444÷445 (2)	−129÷−134	88÷91	24
ID01155.3p-miR	444÷450 (3)	−125÷−134	91÷97	22
ID01641.3p-miR	444÷448 (2)	−136	91	24
ID01702.3p-miR	444÷450 (3)	−138÷−144	86÷92	25
ID01804.3p-miR	438÷444 (3)	−138÷−144	87÷91	25
ID01873.3p-miR	444÷447 (2)	−121÷−123	92÷94	21
ID02187.5p-miR	439÷445 (2)	−123	89	23
ID02770.5p-miR	462	−115	92	20
ID02890.3p-miR	458	−119	89	23
ID03367.5p-miR	441÷453 (2)	−115	92	20
		***SCAP***		
ID00061.3p-miR	102÷114 (5)	−125÷−132	91÷95	22
ID00296.3p-miR	99÷106 (5)	−140÷−144	89÷92	25
ID00756.3p-miR	105÷106 (2)	−123	89	23
ID01041.5p-miR	108	−129	88	24
ID01403.5p-miR	107	−121	89	23
ID01641.3p-miR	102÷108 (4)	−132÷−134	89÷90	24
ID01652.3p-miR	112	−125	89	23
ID01702.3p-miR	105÷112 (5)	−138÷−144	92÷96	24
ID01804.3p-miR	109	−134	91	23
ID01873.3p-miR	108	−125	95	21
ID02294.5p-miR	101	−129	88	24
ID03151.3p-miR	103	−115	93	20
ID03367.5p-miR	108÷111 (2)	−117	93	20
miR-3960	104÷106 (2)	−117	93	20

*In the Table 1 and other Tables in parentheses indicate the number of miRNAs binding sites.*

**FIGURE 1:**
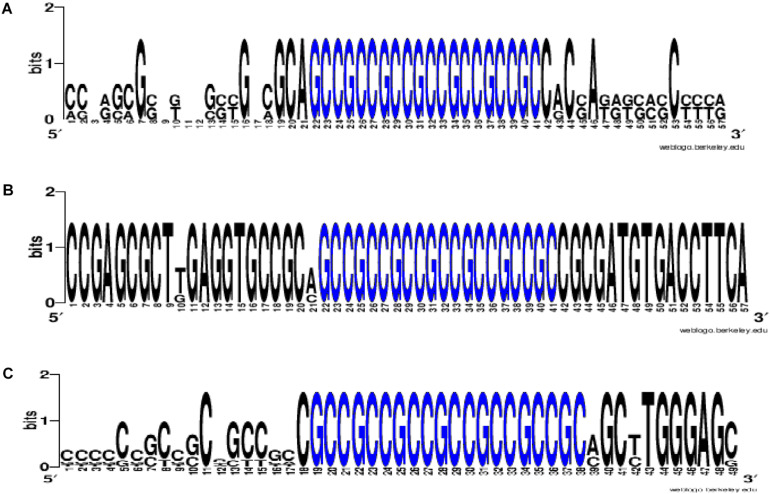
The WebLogo schemes (https://weblogo.berkeley.edu/logo.cgi) of nucleotide sequences for BS clusters in mRNA of *GAS6* **(A)**, *NFE2L2* **(B)**, *SCAP* **(C)** orthological genes. Nucleotides of BS clusters are highlighted in blue. Nucleotides of BS clusters mRNA of *GAS6 Ggo* (*Gîrillà gîrillà*), *Hsa* (*Homo sapiens*), *Mmu* (*Macaca mulatta)*, *Pab* (Pongo abelii), *Pan* (*papio anubis*), *Ptr* (*pan troglodytes*); nucleotides of BS clusters mRNA of *NFE2L2 Ggo*, *Hsa*, *Pab*, *Pan*, *Ptr*; nucleotides of BS clusters mRNA of *SCAP Ggo*, *Hsa*, *Mmu*, *Pab*, *Pan*, *Ptr*.

To prove that the identified clusters are reliable, on the example of *GAS6* gene showed that the cluster exists in the mRNA of orthological genes and is conservative in its nucleotide composition (Figure 1). There are mainly new miRNAs in the clusters of BSs in 5′ UTR region of mRNA of *GAS6, NFE2L2* and *SCAP* genes. In mRNA of *GAS6* gene the cluster includes 20 new miRNA and one old miRNA, in mRNA of *NFE2L2* gene the cluster contains 15 BSs of new miRNA; in mRNA of *SCAP* gene there is a cluster for binding of 13 new and one old miRNA (Table 1). In the other 11 target genes, there are 28 new and one old miRNAs are linked in 5′ UTR region of mRNA (Supplementary Table S2). These data suggest that the use of new miRNAs significantly increased the number of effective gene expression modulators.

Some miRNAs have multiple BSs: ID00061.3p-miR, ID01155.3p-miR, ID01702.3p-miR, ID01804.3p-miR, ID02187.5p-miR, ID00296.3p-miR, ID01155.3p-miR by three, ID01641.3p-miR by four, ID00061.3p-miR, ID00296.3p-miR, ID01702.3p-miR by five BSs. These miRNAs are recommended as associations for the diagnosis of atherosclerosis considering the high free energy interaction with mRNA of candidate genes. ID01935.5p-miR has a complete complementarity interaction with mRNA of *NFE2L2* gene, which suggests that ID01935.5p-miR and *NFE2L2* association should be recommended as a marker. These miRNA correlations clearly demonstrate the predominant influence of new miRNAs on gene expression with BS in 5′ UTR. Consequently, researchers actually do not receive information about the regulation of gene expression by new miRNAs, the effect of which is high when only the old miRNAs are studied.

The following circumstances need to be considered when evaluating the effects of many miRNAs in a cluster of BSs in mRNA. Primarily, miRNA will bind with higher free energy interaction, so quantitative interaction characteristics are needed. The ratio of miRNA concentrations between alternative miRNAs has a significant importance (miRNAs, which bind in the cluster) and especially in relation to the concentration of mRNA. Obviously, the total concentration of alternative miRNAs should be lower than the mRNA concentration, otherwise the protein will not be synthesized. A decrease of concentration of any miRNA below mRNA concentration will not affect protein synthesis. At the same time, an increase of one miRNA above mRNA concentration can completely suppress gene expression. The mRNA of many genes contains clusters of BSs of several similar miRNAs. In this way, nature has optimized the expression of several genes under common miRNA control. It is necessary to consider that many genes are expressed in cells of different tissues to different degrees, which may affect the dependence of protein synthesis from miRNA. However, a large number of binding miRNA may not allow the target gene expression to increase significantly. Many miRNAs have been found in the blood and serum in combination with AGO proteins and within exosomes, which indicates their capability to circulate freely throughout the body and to reach many organs and tissues. It is necessary to remember that the synthesis of intronic miRNAs depends on the expression of host gene, while the synthesis of other miRNAs is made from transcripts of intergenic regions. The reasons for changes in the expression of such miRNA in case of disease should be known. There are known changes of miRNA expression by several orders. Considering the above information, the reports are perceived cautiously that some researchers have been able to detect marker miRNA in specific diseases.

The above examples of presence of BS clusters in mRNA of some genes for several miRNAs suggest that such genes are expressed under the common control of the miRNA group and, consequently, these genes form a network of interconnected genes controlling key metabolic processes. To confirm the reliability of found miRNA and their BSs, it is possible to use data about the presence of such BSs or their clusters in orthological genes (Figure 1).

With the given examples we show the inadequacy of miRNA BSs establishment by programs based only on a miRNA seed sequence. The whole miRNA nucleotide sequence is important, which is confirmed by the conservation of miRNA nucleotide sequences and corresponding BS during millions of years. The nucleotide sequences of miRNA and their BSs have been conserved in the mRNA genes of animal and plant organisms over 10s of millions of years of evolution (Bari et al., 2013; Ivashchenko et al., 2013, 2014b; Yurikova et al., 2019).

ID00061.3p-miR, ID00296.3p-miR, ID00457.3p-miR, ID00522.5p-miR, ID01041.5p-miR, ID01155.3p-miR, ID01641.3p-miR, ID01702.3p-miR, ID01804.3p-miR, ID01873.3p-miR, ID02187.5p-miR and ID03367.5p-miR interact not only with *GAS6* mRNA but also with *NFE2L2* mRNA, where they form a large cluster together with other miRNAs. The cluster of miRNA BS in the *NFE2L2* mRNA is one of the largest clusters of miRNAs associated with atherosclerosis-related genes in which BS are formed in the 5′ UTR. The cluster consists of 14 different miRNAs, ranging from the 438 to 482 nt positions, with a size of 45 nt. The total size of all binding sites is 669 nt, resulting in a degree of compaction of 15. Due to compaction, this length of BS is a small fraction of the 5′ UTR length of 555 nt. A complete complementarity interaction of ID01935.5p-miR with *NFE2L2* mRNA was identified. Eight miRNAs interact with *NFE2L2* mRNA with a free energy of more than −130 kJ/mole. These associations are recommended as valuable markers of atherosclerosis. The above examples of associations of miRNAs and target genes show that determining the expression level of one or more miRNA without monitoring target gene expression will not provide adequate data on the specific association of these miRNAs with the disease.

**FIGURE 2:**
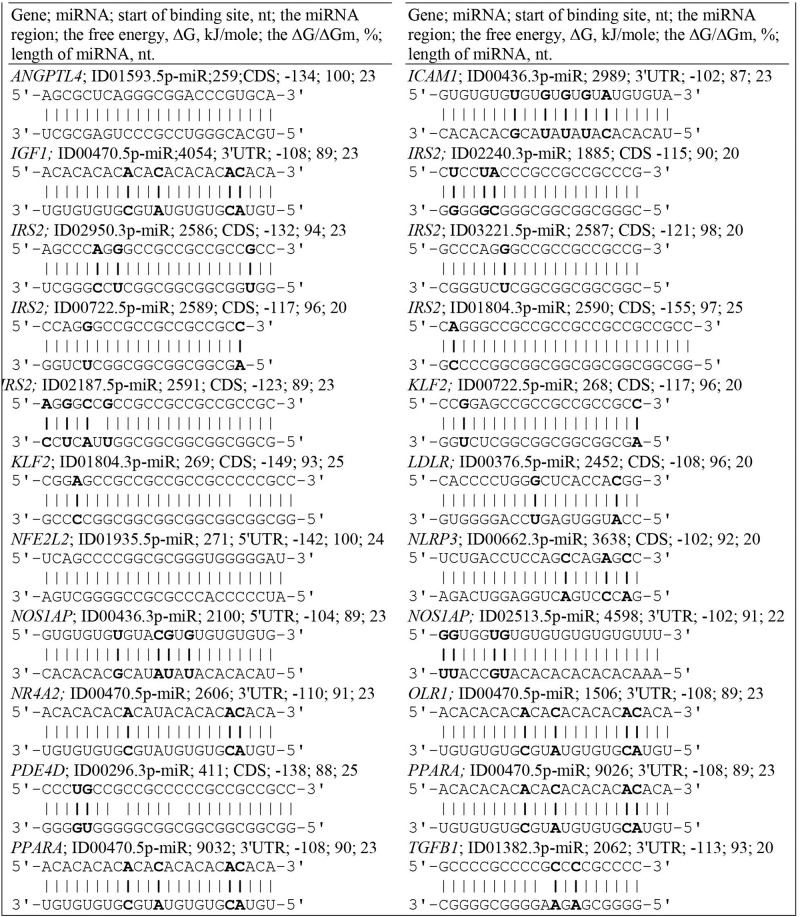
Convincing evidence of the effectiveness of the MirTarget program in determining the characteristics of miRNA binding to the 5′ UTR, CDS and 3′ UTR of mRNA. The diagrams show the interaction of nucleotides along the entire length of the miRNA in the BS of the target mRNA and the characteristics of this interaction. Non-canonical base pairs are in bold.

The *ADAM10* mRNA contains BS for ID02761.3p-miR with overlapping nucleotide sequences (Supplementary Table S2). The *ADCY9* mRNA is characterized by the presence of two clusters, each of which is formed by two single BS. In addition to this gene, clusters were identified in the following mRNAs: *IRS2* and *PIN1*, which contain BS for two miRNAs. The *PNPLA3* and mRNAs have a cluster consisting of three single BS. The *CXCL12* mRNA contains a cluster of four different miRNAs, of which ID02036.3p-miR, ID01293.5p-miR, ID00417.3p-miR and ID02066.5p-miR have two BS. The cluster starts at 65 nt and ends at 93 nt, with a length of 29 nt and an average ΔG/ΔGm of 93%. The sum of the BS lengths of these miRNAs was 170 nt and was six times longer than the cluster length. Due to this compaction, all BS were located in a 5′ UTR with a length of 92 nt. The *MMP2* mRNAs contained a cluster of six miRNA BS with a total length of 136 nt and a 5′ UTR length of 311 nt. The *PNPLA3* mRNA contains a cluster of three miRNA BS. Among the clusters in the 5′ UTRs of the target genes, one can distinguish the *SCAP* gene cluster formed by BS from 14 miRNAs from the 99 nt to the 136 nt position, in which both single BS and multiple BS are found, more specifically, seven multiple sites and seven single sites (Table 1). Of the 14 miRNAs with the exception of ID00756.3p-miR, ID01403.5p-miR, ID01652.3p-miR and ID03151.3p-miR all were in the group of miRNAs binding to the *GAS6* mRNA. Furthermore, ID00061.3p-miR, ID00296.3p-miR, and ID01702.3p-miR had five BS each, which significantly increased the likelihood of their binding compared to that of competing miRNAs. The total length of miRNA BS was 735 nt, and the degree of compaction was 19. Without compaction, these sites could not be located in this 255 nt long 5′ UTR. This cluster is characterized by a maximum ΔG of −144 kJ/mole for the BS of ID00296.3p-miR and ID01702.3p-miR. Of all the interactions of miRNAs and the 5′ UTRs of target mRNAs, five had a free energy of more than −130 kJ/mole. Convincing evidence of the effectiveness of the MirTarget program in determining the characteristics of miRNA binding to the 5′ UTR, CDS and 3′ UTR of mRNA is given in Figure 2. The diagrams show the interaction of nucleotides along the entire length of the miRNA in the BS of the target mRNA and the characteristics of this interaction.

### Characteristics of miRNA Interactions With the CDS of mRNAs of Atherosclerosis Candidate Genes

Most genes contained more one BS for one miRNA in the CDS of mRNAs. There were some genes with several BS or multiple BS where clusters could form. It is worth noting that *IRS2* is characterized by the presence of three clusters and a large number of BS compared to other genes. This suggests that this gene is more susceptible to regulation by miRNA (Table 2). The first cluster was composed of 25 different miRNAs, 13 of which had two or more BS (ID00061.3p-miR, ID00457.3p-miR, ID00756.3p-miR, ID01155.3p-miR, ID01702.3p-miR, ID01804.3p-miR, ID01873.3p-miR, ID01879.5p-miR ID02064.5p-miR, ID02187.5p-miR, ID03229.5p-miR, ID03367.5p-miR, miR-3960), and the rest were composed of single BS. The cluster was 41 nt long, starting at 2586 nt and ending at 2626 nt. The total length of the BS in the cluster was 1114 nt, while the degree of cluster compaction was 28. The 2-nd cluster was formed by single BS of seven miRNAs. The 3-day cluster consisted of the BS of nine different miRNAs. ID00296.3p-miR and ID01702.3p-miR had three binding sites. In general, the cluster was 41 nt long, extending from position 4304 to 4344 nt. The total length of all the BS of the three clusters was 1607 nt, which is 40% of the total CDS length of 4017 nt. Due to compaction, the total length of all clusters is 110 nt, that is, only 2.7%. Thirteen miRNAs interacted with the *IRS2* mRNA with a free energy of more than −130 kJ/mole, which gives reason to recommend these interactions as diagnostic markers of atherosclerosis. Each of the three clusters in the MR of the orthologous IRS2 genes encoded different highly homologous oligopeptides (Supplementary Table S3).

**TABLE 2:** Characteristics of miRNAs interaction in the CDS of mRNA of *IRS2* and *KLF2* genes.

miRNA	Start of site, nt	ΔG, kJ/mole	ΔG/ΔGm, %	Length, nt
***IRS2***				
ID00061.3p-miR	2589÷2601 (4)	−125÷−136	91÷98	22
ID00296.3p-miR	2589	−136	86	25
ID00457.3p-miR	2592÷2595 (2)	−123÷−132	91÷97	22
ID00522.5p-miR	2589	−127	91	23
ID00756.3p-miR	2587÷2593 (2)	−123÷−125	89÷91	23
ID01041.5p-miR	2592	−138	94	24
ID01155.3p-miR	2595÷2601 (4)	−125÷−136	91÷98	22
ID01574.5p-miR	2591	−127	90	23
ID01702.3p-miR	2589÷2599 (4)	−136÷−140	90÷93	25
ID01778.3p-miR	2594	−134	90	24
ID01804.3p-miR	2589÷2596 (5)	−140÷−155	88÷97	25
ID01873.3p-miR	2592÷2595 (2)	−123	94	21
ID01879.5p-miR	2594÷2600 (2)	−123	91	22
ID01895.5p-miR	2586	−134	90	24
ID02052.5p-miR	2589	−132	89	24
ID02064.5p-miR	2594÷2603 (4)	−129÷−132	90÷91	23
ID02187.5p-miR	2591÷2593 (2)	−123÷−125	89÷91	23
ID02294.5p-miR	2594	−134	91	24
ID02950.3p-miR	2586	−132	94	23
ID03064.3p-miR	2589	−140	92	24
ID03221.5p-miR	2587	−121	98	20
ID03229.5p-miR	2590÷2593 (2)	−121	90	22
ID03305.5p-miR	2589	−115	95	20
ID03367.5p-miR	2592÷2598 (3)	−117	93	20
miR-3960	2594÷2597 (2)	−115	92	20
ID01702.5p-miR	3602	−138	88	25
ID01574.5p-miR	3603	−127	90	23
ID01804.3p-miR	3599	−140	88	25
ID01106.5p-miR	3603	−129	87	24
ID02229.3p-miR	3605	−125	95	21
ID02499.3p-miR	3605	−121	93	21
miR-3960	3604	−115	92	20
ID00061.3p-miR	4316	−125	91	22
ID00296.3p-miR	4304÷4310 (3)	−134÷−144	85÷92	25
ID00457.3p-miR	4319	−123	91	22
ID01155.3p-miR	4316	−125	91	22
ID01641.3p-miR	4310	−134	90	24
ID01702.3p-miR	4310÷4316 (3)	−134÷−136	89÷90	25
ID01804.3p-miR	4313	−138	87	25
ID02064.5p-miR	4321	−129	90	23
ID02187.5p-miR	4314	−125	91	23
***KLF2***				
ID00061.3p-miR	274	−129	94	22
ID00457.3p-miR	271	−127	94	22
ID00722.5p-miR	268	−117	96	20
ID01155.3p-miR	274	−129	94	22
ID01377.3p-miR	273	−121	95	20
ID01445.3p-miR	265	−115	92	20
ID01702.3p-miR	266	−136	90	24
ID01804.3p-miR	269	−149	93	23
ID01895.5p-miR	264	−140	94	24
ID02187.5p-miR	270	−123	89	23
ID02950.3p-miR	265	−134	95	23
ID03221.5p-miR	266	−121	98	20
ID03367.5p-miR	271	−115	92	20
miR-4787-5p	269	−123	92	22

Clusters consisting of single miRNA BS were found in the following genes (Supplementary Table S4): two BS in *ACE* with an average ΔG of −128 kJ/mole; five BS in *ADRB3* for five miRNAs with an average ΔG of −115 kJ/mole and BS in *FASLG* for ID00061.3p-miR, ID00296.3p-miR, ID01641.3p-miR, and ID01702.3p-miR. *SIRT1* contained two sites for ID03332.3p-miR and single BS for ID00278.3p-miR and ID00811.3p-miR, with an average ΔG of −129 kJ/mole.

*TBC1D10B* gene is involved in the functioning of cardiovascular endothelial cells (Li et al., 2017) and it is a target of miR-762 with fully complementary binding (Supplementary Table S4). This site encodes the APAPAPAPAPAPA oligopeptide, with the flanking oligopeptides AWVPGSAQTS and VTGSTVVVLTL (Supplementary Table S5).

MRNA of *CDKN1C* gene (Rodriguez et al., 2007) contains three clusters consisting of both single and multiple BS. The first cluster consists of two BS for miR-762 and ID03129.3p-miR and has a size of 36 nt, extending from 738 to 753 nt. The second cluster is composed of 17 miR-762 BS, 3 ID00099.3p-miR BS, and 1 ID02682.5p-miR BS. ID00036.3p-miR, ID01075.3p-miR and ID00411.5p-miR cover a region of 31 nt, extending from the 888 nt position to 918 nt with an average ΔG value of −125 kJ/mole. The mRNA of the candidate gene *CDKN1C* has 17 sequentially located binding sites for miR-762 that encode the oligopeptide (AP)18, which indicates a strong dependence of gene expression on this miRNA (Supplementary Table S4). Orthologous primate genes (Supplementary Table S6) encode similar oligopeptides with an AP dipeptide number up to 33. In all primate cases, miR-762 BSs are located between the conserved flanking nucleotide sequences that encode the conserved AAPVAVAVLA and DAAPQESAEQ oligopeptides (Supplementary Table S6). These animals can be used to study the role of miR-762 in the development of atherosclerosis. A similar type of organization of miRNA binding multiple sites has been found in the mRNAs of many genes involved in the development of cardiovascular and other diseases (Kondybayeva et al., 2018; Aisina et al., 2019; Yurikova et al., 2019).

*KLF2* is characterized by the presence of two clusters consisting of the BS for 14 and 2 different miRNAs. The first of these clusters is formed by single BS starting from the 264 to 296 nt position with a size of 33 nt (Table 2). The total length of all BS is 305 nt. The degree of compaction of the binding sites in the cluster is nine. The second cluster of this gene is formed only by two single BS. In the CDS of *KLF2* mRNA BS were detected for ID00061.3p-miR, ID00457.3p-miR, ID01155.3p-miR, ID01702.3p-miR, ID01804.3p-miR, ID02187.5p-miR, ID03367.5p-miR which have BS in the 5′ UTR of *GAS6* mRNA. This indicates an overall control of these genes expression by these miRNAs. The maximum ΔG/ΔGm value (100%) among these genes was detected at the ID01593.5p-miR BS, which interact with the *ANGPTL4* mRNA.

*PDE4D* formed two clusters. The first cluster contained the BS for ID00061.3p-miR, ID01155.3p-miR, ID03064.3p-miR, miR-3960 and ID01702.3p-miR, which had three BS with a free energy value of −134 kJ/mole. ID01641.3p-miR, miR-3960 and ID01702.3p-miR BS were found in the second cluster. The size of the first cluster was 34 nt, extending from position 336 to 369 nt. The second cluster consisted of five multiple BS and eight single BS; the cluster size was 49 nt, extending from 391 to 439 nt. The total length of miRNA BS was 491 nt, and the degree of compaction was 10 (Supplementary Table S4).

There were 39 BS for new miRNAs and two BS for old miRNA in *IRS2* mRNA. Two clusters of binding sites in the *KLF2* mRNA interacted with 14 new miRNAs and one old miRNA. In addition, there were 15 BS for new miRNA and two BS for miR-3960 in the *PDE4D* mRNA. A total in CDS mRNA of 40 target genes BS were identified for 148 new miRNAs and 32 old miRNAs.

Some miRNAs had more than two BS: ID01641.3p-miR, ID02064.5p-miR, ID02187.5p-miR, ID02770.5p-miR, ID03324.3p-miR, ID03367.5p-miR – 3 BS; ID00296.3p-miR, ID01377.3p-miR, ID01804.3p-miR, miR-3960 – 4 BS; ID00457.3p-miR, ID01155.3p-miR – 5 BS and ID00061.3p-miR, ID01702.3p-miR – 6 BS. Most of them had free energy interaction above −130 kJ/mole.

Three clusters of miRNA BS in CDS mRNA of orthologic genes encode different oligopeptides because they have different reading frames. Oligopeptides encoded by BS are clearly defined by conservative oligopeptides, which flank oligopeptides encoded by BS. Apparently, flanking oligopeptides carry out the important functional role of a protein, and oligopeptides coded by BS in the first and second clusters change for the reason of an acceptability of incomplete complementary interactions of miRNA and mRNA.

### Characteristics of miRNA Interactions in the 3′ UTRs of mRNAs of Atherosclerosis Candidate Genes

The 40 genes involved in the development of atherosclerosis were characterized by BS in the 3′ UTRs. A feature of miRNA interactions with atherosclerosis candidate genes is the presence of BS for miR-466, ID00436.3p-miR, and ID01030.3p-miR in their mRNAs (Table 3 and Supplementary Table S7). The BS of these miRNAs can be single or multiple and could form a cluster. For example, *CD36* mRNA contains six BS for ID00436.3p-miR and five BS for miR-466 and ID01030.3p-miR. *FASLG* mRNA contains six BS for miR-466 and ID00436.3p-miR and five BS for ID01030.3p-miR. The mRNA of the *FLT1* gene contains eight BS for miR-466 and seven BS for ID00436.3p-miR and ID01030.3p-miR. Another feature of candidate genes is the presence of clusters of binding sites for miR-5095 and miR-619-5p in their mRNA. There are two clusters of BS of miR-5095 and miR-619-5p located 12 nucleotides apart in *IL18* mRNA. In these clusters, the start sites of miR-5095 and miR-619-5p BS are located six nucleotides apart, which is probably due to their common origin. In the mRNAs of *CD36* and *ADAM17* completely complementary BS with miR-619-5p and BS between the mRNA of *IL18* with miR-5096 were revealed. The BS of miR-1273f and miR-1273e form a cluster in the mRNAs of *IGF1* gene (Supplementary Table S7). The 3′ UTRs of the mRNAs of many candidate genes contain only single miRNA BS that do not form clusters: *ABO, ACE, ADAM17, ADAM33, ANGPT2, APOL1, CD59, FADS2, FOXP3, GPR132, HNF1A, IGF1R, LDLR, LPCAT3, NOS1AP, PNPLA3, PPARA, SOAT1, SOCS3, TFPI*, *TNC*, and *ZBTB46*. Some genes are characterized by clusters consisting of two single BS: *BRCA1, F11R, IGF1, ITGA2*, and *TNFSF10.* Some genes were characterized by mixed clusters formed by both single and multiple BS. *NR4A2* had a cluster consisting of both single BS of ID00470.5p-miR and multiple BS of ID02299.5p-miR (Supplementary Table S7).

**TABLE 3:** Characteristics of miRNAs interaction in the 3′ UTR of mRNA of atherosclerosis candidate genes.

Gene	miRNA	Start of site, nt	ΔG, kJ/mole	ΔG/ΔGm, %	Length, nt
*ADRB3*	ID02868.3p-miR	2442	−115	92	23
	miR-466	2451	−110	95	23
	ID00436.3p-miR	2456	−108	93	23
	ID01030.3p-miR	2456÷2462 (2)	−113	93	23
*CD36*	miR-466	3530÷3538 (5)	−106÷−108	91÷93	23
	ID00436.3p-miR	3533÷3543 (6)	−104	89	23
	ID01030.3p-miR	3529÷3539 (5)	−108	89	23
	ID01727.5p-miR	3542	−106	91	23
	miR-619-5p	4041	−121	100	22
	miR-5096	4107	−104	92	21
	miR-619-5p	4168	−117	96	22
	miR-5585-3p	4175	−108	93	22
*FASLG*	ID02868.3p-miR	1602	−113	90	23
	miR-466	1603÷1613 (6)	−106÷−108	91÷93	23
	ID00436.3p-miR	1604÷1614 (6)	−104÷−106	89÷91	23
	ID01030.3p-miR	1604÷1612 (5)	−108	89	23
*FLT1*	miR-466	6910÷6936 (8)	−106÷−108	91÷93	23
	ID00436.3p-miR	6913÷6925 (7)	−104	89	23
	ID01030.3p-miR	6909÷6923 (7)	−108÷−110	89÷91	23
*ICAM1*	miR-466	2988	−106	91	23
	ID01030.3p-miR	2987	−108	89	23
	ID01360.3p-miR	3022	−104	91	21
	ID00367.5p-miR	3025	−110	90	22
	miR-1273g-3p	3031	−115	98	21
*IL18*	miR-5095	811÷823 (2)	−110	95	21
	miR-619-5p	817÷829 (2)	−119		22
	miR-5096	890÷903 (4)	−102÷−113	91÷100	21
*PLA2G7*	miR-466	1643÷1651 (5)	−106÷−108	91÷93	23
	ID00436.3p-miR	1646÷1654 (5)	−104	89	23
	ID01030.3p-miR	1646÷1652 (4)	−108	89	23
*PPARGC1A*	miR-466	2806÷2822 (2)	−106	91	23
	ID00436.3p-miR	2809÷2825 (3)	−104÷−108	89÷93	23
	ID01030.3p-miR	2811	−115	95	23
	ID01727.5p-miR	2824	−104	89	23

*FASLG* was characterized by a cluster formed from a miRNA with single BS (ID02868.3p-miR) and three miRNAs with multiple BS consisting of six BS for miR-466, five for ID01030.3p-miR and six for ID00436.3p-miR, extending from 1602 to 1637 nt with an average ΔG value of −108 kJ/mole. In the 3′ UTR of *FLT1*, there was a cluster of three multiple BS consisting of seven BS for ID01030.3p-miR, seven for ID00436.3p-miR and eight for miR-466, extending from 6909 to 6959 nt and with an average ΔG value of −107 kJ/mole. *IL18* had a cluster of two miR-5095 and two miR-619-5p sequences, with a size of 41 nt and an average ΔG value of −114 kJ/mole. *MTHFR* mRNA contained one cluster, consisting of ID01811.5p-miR, miR-5095 and miR-619-5p with an average ΔG value of −114 kJ/mole. *PPARGC1A* contained a cluster formed by four different miRNAs, including two miR-466 BS and three ID00436.3p-miR, ID01030.3p-miR, and ID01727.5p-miR sequences. The size of this cluster was 43 nt, extending from 2806 to 2848 nt. There were some genes with more than one cluster. The *PLA2G7* mRNA had a cluster that consisted of five miR-466, four ID01030.3p-miR and five ID00436.3p-miR sites and a length of 35 nt, extending from 1643 to 1677 nt, with an average ΔG value of −106 kJ/mole.

*ICAM1* was characterized by the presence of two clusters, the first of which consisted of two single BS (ID01030.3p-miR and miR-466), extending from 2987 to 3011 nt. The second cluster consisted of three BS of different miRNAs. ID00470.5p-miR formed BS in *OLR1* and *NR4A2* mRNAs. The cluster of miR-1273f and miR-1273e BS was formed in *IGF1* mRNA.

There were miRNAs that had BS for more than one mRNA. The unique miR-619-5p had BS in the mRNAs of *ADAM17, ADAM33, APOL1, BRCA1, F11R, IL18, ITGA2, LDLR, MTHFR, PNPLA3, SOAT1*, and *TNFSF10*. ID00436.3p-miR and ID01030.3p-miR formed clusters that consisted of multiple BS for *ADRB3, F11R, FASLG, FLT1, PLA2G7*, and *PPARGC1A* mRNA. ID01727.5p-miR had BS for *NOS1AP* and *PPARGC1A* mRNAs.

The *CD36* mRNA cluster, consisting of BS to two different miRNAs, miR-619-5p and miR-5585-3p, extended from 4168 to 4197 nt. The *CD36* mRNA cluster consisted of both single BS and multiple BS. In general, the cluster consisted of five ID01030.3p-miR, five miR-466, six ID00436.3p-miR and one ID01727.5p-miR sequences. The size was 38 nt, extending from 3529 to 3566 nt, with an average ΔG value of −106 kJ/mole.

Associations of miRNA and target genes having BS in 3′ UTR mRNA are very different from those in 5′ UTR and CDS. In the 3′ UTR mRNA there are no clusters of BS over four miRNAs. The number of old and new miRNAs is comparable. The total number of binding sites of new miRNA is 57 and 63 old miRNA per mRNA of 40 target genes. This is probably due to the fact that the GC content of old miRNAs is comparable to that of 3′ UTRs, while in 5′ UTR and CDS it is about 10% higher. In the new miRNA GC content is comparable to that in 5′ UTR and CDS, so the number of BS for new miRNA in these regions of mRNA is much larger.

The choice of miRNA-gene associations is difficult to offer as miRNAs such as miR-619-5p, miR- 5095-, miR-5096- and miR-5585-3p, which have targets in many genes and most likely play the role of stabilizers of protein expression. Similar to miR-466, ID00436.3p-miR and ID01030.3p-miR, which have multiple BS in the mRNA of many genes, which also gives them the role of stabilizing the expression of their targets. A significant increase in the concentration of any these miRNAs will lead to numerous metabolic disorders and to different diseases consequently. If about 100–200 key miRNAs are identified with miRNA-chips, the range of candidate genes will be significantly reduced because with bioinformatic approaches it could be done easily and quickly.

## Discussion

Since it would be advisable to have lower costs for the diagnosis of the disease, it is desirable to minimize the number of associations of miRNA and target genes. However, in the case of polygenic diseases, such methods could be difficult to develop because it is not known which gene and which miRNA cause a particular patient’s disease. Therefore, now it is necessary to select a list of priority genes for diagnosis and then check which miRNA could influence protein expression. With the MirTarget program, this is easily to install and, if alternative miRNAs are involved, it is also simple to identify miRNAs competitiveness. The results of the present research demonstrate the effectiveness of this approach.

It was found that the BS of some miRNAs formed clusters in the 5′ UTR and CDS sequences of mRNA, resulting in competition between these miRNAs for binding to mRNA and, accordingly, for suppressing the expression of the target gene. The quantitative characteristics of miRNA BS with mRNA make it possible to predict which miRNAs can more efficiently bind to mRNA at equal miRNA concentrations. At different concentrations of these miRNAs, kinetic equations can be used to predict their suppressive effect on the expression of the target gene. The revealed associations of several miRNAs that bind to mRNAs of different candidate genes make it possible to predict the effect of these miRNAs on the corresponding genes, which can be expressed to different degrees. For example, *GAS6, NFE2L2*, and *IRS2* are targets of several identical miRNAs and bind with these miRNAs depending on their concentrations in the cell. In cells of different tissues, the ratio of miRNA and target gene concentrations will be different, and the effect of the miRNAs on these target genes will also be different. This brief discussion of the different interactions of miRNAs and candidate genes under different circumstances shows the complexity of these networks and begins to paint a picture of the interactions of miRNA groups with groups of target genes. Without this assumption, it is difficult to predict or evaluate the involvement of a single miRNA or a single gene in the development of a disease. From the identified interactions, it is necessary to choose those that control more genes and miRNAs and reflect the development of atherosclerosis. A number of mRNAs and miRNAs strongly interacting with each other are proposed in this study. For example, *PDE4D* and ID00296.3p-miR; *SCAP* and ID00296.3p-miR and ID01702.3p-miR; *ADCY9* and ID00296.3p-miR; *IRS2* and ID01702.3p-miR, ID03064.3p-miR, ID01804.3p-miR, and ID00296.3p-miR; *NFE2L2* and ID01935.5p-miR, ID00296.3p-miR, ID01702.3p-miR, and ID01804.3p-miR. In addition, miRNAs that bind with mRNAs of candidate genes with complete complementarity are of interest. Within addition to this condition, it is necessary to consider miRNAs interacting with a large number of genes, since such miRNAs can simultaneously control the expression of many genes. Genes that are regulated by a large number of miRNAs can also be considered as effective markers. Unfortunately, our knowledge is still insufficient to determine which candidate genes play a key role in the development of the disease, and it is not known which miRNAs are the most likely signals for the development of atherosclerosis. However, one thing is obvious – it is necessary to simultaneously study miRNA and gene interactions in order to improve the likelihood of obtaining a valid result. From the established interactions, it is first necessary to consider the interactions between candidate genes with multiple miRNAs that form clusters of BS and miRNA interactions with high free energy. For example, *IRS2* is the target of 41 miRNAs with BS located in the CDS, of which ID00061.3p-miR, ID00296.3p-miR, ID00457.3p-miR, ID01041.5p-miR, ID01155.3p-miR, ID01641.3p-miR, ID01702.3p-miR, ID01778.3p-miR, ID01804.3p-miR, ID01895.5p-miR, ID02052.5p-miR, ID02064.5p-miR, ID02950.3p-miR, and ID03064.3p-miR have free energy interactions above −130 kJ/mole. The mRNA of *GAS6* interacts with 21 miRNAs, of which those with ID00296.3p-miR, ID01041.5p-miR, ID01106.5p-miR, ID01641.3p-miR, ID01702.3p-miR, ID01804.3p-miR, ID02084.3p-miR, and ID02294.5p-miR are more efficient. Associations of this type were observed for *NFE2L2, ADCY9, PNPLA3, SCAP*, *KLF2* and *PDE4D*, which bind to many miRNAs with high free energy. Another type of association is the interaction of one or more miRNA with many genes. For example, miR-466, ID00436.3p-miR and ID01030.3p-miR bind with the mRNAs of *ADRB3, CD36, FASLG, FLT1, PLA2G7*, and *PPARGC1A*. In addition, miR-619-5p targets several candidate atherosclerosis genes: *CD36, IL18, ADAM17, ADAM33, APOL1, BRCA1, F11R, ITGA2, LDLR, MTHFR, PNPLA3, SOAT1*, and *TNFSF10*. The mRNA of *ANGPTL4* is fully complementary with ID01593.5p-miR; the mRNAs of *ADAM17* and *CD36* with miR-619-5p; the mRNA of *IL18* with miR-5096; and the mRNA of *NFE2L2* with ID01935.5p-miR.

Here, miRNA interactions with candidate atherosclerosis genes have been established, which consist of one gene and several miRNAs, one gene and one miRNA, one miRNA and several genes, and two or more miRNAs with two or more candidate genes. The revealed cluster organization of miRNA BS in mRNA candidate genes contributes to a more accurate diagnosis of the participation of competing miRNAs in the development of atherosclerosis.

## Data Availability Statement

The datasets presented in this study can be found in online repositories. The names of the repository/repositories and accession number(s) can be found in the article/ Supplementary Material.

## Author Contributions

AI and DM conceived of the study, drafted the manuscript, and gave final approval of the version to be published. SL, DA, AP, and AR conceived of the study and drafted the manuscript. All authors made substantial contributions to acquisition of data, to interpretation and modification of the data, were involved in subsequent rounds of revisions, and read and approved the final manuscript.

## Conflict of Interest

The authors declare that the research was conducted in the absence of any commercial or financial relationships that could be construed as a potential conflict of interest.

## Footnotes

1 http://mirbase.org

2 http://www.ncbi.nlm.nih.gov
