# Supplementary material for: In silico Prediction of miRNA Interactions With Candidate Atherosclerosis Gene mRNAs
Source: Front Genet. 2020 Nov 4;11:605054. doi: 10.3389/fgene.2020.605054 (PMC7672156; doi:10.3389/fgene.2020.605054)
Supplement: Supplementary file 1 [file Table_1.DOCX]

**Table S1.** The database of candidate genes responsible for the development of atherosclerosis 68 genes

| Gene | ID | PMID | Gene | ID | PMID |
| --- | --- | --- | --- | --- | --- |
| *ABO* | 28 | 26924317 | *IL18* | 3606 | 31281442 |
| *ACE* | 1636 | 23540412 | *IRS2* | 8660 | 23099119 |
| *ADAM10* | 102 | 31632389 | *ITGA2* | 3673 | 20485444 |
| *ADAM17* | 6868 | 28062509 | *KLF2* | 10365 | 31222221 |
| *ADAM33* | 80332 | 24699314 | *LDLR* | 3949 | 31778654 |
| *ADAMTS13* | 11093 | 29885460 | *LPCAT3* | 10162 | 30705887 |
| *ADAMTS7* | 11173 | 31679296 | *MMP2* | 4313 | 31733453 |
| *ADCY9* | 115 | 29674325 | *MTHFR* | 4524 | 29501539 |
| *ADRB3* | 155 | 24658877 | *NFE2L2* | 4780 | 30768972 |
| *ANGPT2* | 285 | 30688091 | *NOS1AP* | 9722 | 19943157 |
| *ANGPTL4* | 51129 | 30688091 | *NOS3* | 4846 | 30128915 |
| *APLN* | 8862 | 29336478 | *NR4A2* | 4929 | 25089663 |
| *APOB* | 338 | 31669498 | *OLR1* | 4973 | 28146073 |
| *APOE* | 348 | 31792364 | *PDE4D* | 5144 | 20540798 |
| *APOL1* | 8542 | 28298955 | *PIN1* | 5300 | 28986099 |
| *BRCA1* | 672 | 23415688 | *PLA2G7* | 7941 | 29348973 |
| *CD36* | 948 | 29534172 | *PLTP* | 5360 | 28137768 |
| *CD59* | 966 | 24084445 | *PNPLA3* | 80339 | 31721770 |
| *CDC42* | 998 | 25057989 | *PPARA* | 5465 | 27698357 |
| *CDK5* | 1020 | 22753194 | *PPARGC1A* | 10891 | 21042583 |
| *CDKN1C* | 1028 | 17351341 | *RTN4* | 57142 | 24778558 |
| *CXCL12* | 6387 | 31662443 | *SCAP* | 22937 | 30462530 |
| *F11R* | 50848 | 20627246 | *SOAT1* | 6646 | 29567472 |
| *FADS2* | 9415 | 19172737 | *SOCS3* | 9021 | 28217098 |
| *FASLG* | 356 | 15927188 | *SIRT1* | 23411 | 26296466 |
| *FLT1* | 2321 | 21310411 | *TBC1D10B* | 26000 | 28334711 |
| *FOXP3* | 50943 | 29618596 | *THBD* | 7056 | 22232927 |
| *GAS6* | 2621 | 19644365 | *TFPI* | 7035 | 30343349 |
| *GPR132* | 29933 | 29796244 | *TNC* | 3371 | 23433402 |
| *GSTP1* | 2950 | 28215799 | *TNFSF10* | 8743 | 23074332 |
| *HNF1A* | 6927 | 27534721 | *VEGFA* | 7422 | 31669080 |
| *ICAM1* | 3383 | 31553645 | *VWF* | 7450 | 31199942 |
| *IGF1* | 3479 | 27835972 | *UTS2R* | 2837 | 23344731 |
| *IGF1R* | 3480 | 30354209 | *ZBTB46* | 140685 | 29884909 |
