# Supplementary material for: In silico Prediction of miRNA Interactions With Candidate Atherosclerosis Gene mRNAs
Source: Front Genet. 2020 Nov 4;11:605054. doi: 10.3389/fgene.2020.605054 (PMC7672156; doi:10.3389/fgene.2020.605054)
Supplement: Supplementary file 2 [file Table_2.DOCX]

**Table S2.** Characteristics of miRNAs interaction in the 5’UTR of mRNA of atherosclerosis candidate genes

| **Gene** | **miRNA** | **Start of site, nt** | **∆G, kJ/mole** | **G/∆Gm, %** | **Length, nt** |
| --- | --- | --- | --- | --- | --- |
| *ADAM10* | ID02761.3p-miR | 416 | -132 | 89 | 24 |
| *ADCY9* | ID00296.3p-miR | 96 | -140 | 89 | 25 |
|  | ID01641.3p-miR | 96 | -136 | 91 | 24 |
|  | ID02456.3p-miR | 195 | -127 | 90 | 23 |
|  | ID02691.5p-miR | 205 | -123 | 89 | 24 |
| *APLN* | ID02644.3p-miR | 146 | -121 | 97 | 22 |
|  | ID02891.3p-miR | 263 | -123 | 89 | 23 |
| *CDC42* | ID01838.5p-miR | 115 | -121 | 97 | 24 |
| *CDK5* | ID01151.3p-miR | 14 | -121 | 88 | 24 |
|  | ID02541.5p-miR | 93 | -129 | 97 | 22 |
| *CXCL12* | ID02036.3p-miR | 65÷69(2) | -115 | 92 | 20 |
|  | ID01293.5p-miR | 66÷70(2) | -123÷-125 | 91÷92 | 22 |
|  | ID00417.3p-miR | 67÷71(2) | -123 | 94 | 21 |
|  | ID02066.5p-miR | 67÷71(2) | -123÷-129 | 91÷95 | 22 |
| *GSTP1* | ID03331.3p-miR | 91 | -129 | 90 | 23 |
| *IRS2* | ID02344.3p-miR | 66 | -132 | 91 | 24 |
|  | ID01774.5p-miR | 438 | -129 | 90 | 23 |
|  | ID02833.5p-miR | 481 | -121 | 90 | 22 |
|  | miR-4767 | 490 | -127 | 90 | 23 |
| *MMP2* | ID00278.3p-miR | 110 | -123 | 89 | 23 |
|  | ID01310.3p-miR | 113 | -121 | 92 | 22 |
|  | ID03037.3p-miR | 115 | -121 | 90 | 22 |
|  | ID03280.3p-miR | 115 | -121 | 90 | 22 |
|  | ID03345.5p-miR | 124 | -127 | 90 | 24 |
|  | ID03368.3p-miR | 125 | -117 | 89 | 23 |
| *PIN1* | ID01667.3p-miR | 64 | -127 | 97 | 22 |
|  | ID02121.3p-miR | 69 | -129 | 92 | 23 |
| *PNPLA3* | ID03418.3p-miR | 148 | -136 | 96 | 23 |
|  | ID00089.3p-miR | 149 | -136 | 98 | 22 |
|  | ID01641.3p-miR | 152 | -134 | 90 | 24 |
