# Supplementary material for: In silico Prediction of miRNA Interactions With Candidate Atherosclerosis Gene mRNAs
Source: Front Genet. 2020 Nov 4;11:605054. doi: 10.3389/fgene.2020.605054 (PMC7672156; doi:10.3389/fgene.2020.605054)
Supplement: Supplementary file 3 [file Table_3.DOCX]

**Table S3.** Oligopeptides encoded by miRNA binding sites of three clusters found in mRNA coding region of orthologous gene *IRS2*

| Plots of proteins containing oligopeptides encoded by mRNA binding sites of orthologous gene *IRS2*. | Object |
| --- | --- |
| BS cluster 2586-2614nt |  |
| SAPKQILQ**PRAAAAAAAA**TVPPAGPAG | *Рарiо Аnubis* |
| SAPKQILQ**PRAAAAAAAA**-VPSAGPAG | *Hоmо sарiеns* |
| SAPKQILQ**PRAAAAAAA**-TVPPAGPAG | *Mасаса mulаttа* |
| SAPKQILQ**PRAAAAAAA**--VPSAGPAG | *Gоrillа gоrillа* |
| SAPKQILQ**PRAAAAAA**---VPSAGPAG | *Раn раnisсиs* |
| SAPKQILQ**PRAAAAAA**---VPSAGPAG | *Раn trоglоdуtеs* |
| BS cluster 3599-3627nt |  |
| SASPSSS**LQPPPPPPAP**GELYRLPPAS | *Hоmо sарiеns* |
| SASPSSS**LQPPPPPPPP**GELYRLPPAS | *Mасаса mulаttа* |
| SASPSSS**LQPPPPPPPP**GELYRLPPAS | *Раn trоglоdуtеs* |
| SASPSSS**LQPPPPPPPP**GELYRLPPAS | *Gоrillа gоrillа* |
| SASPSSS**LQPPPPPPPP**GELYRLPPAS | *Рарiо Аnubis* |
| BS cluster 4310-4344nt |  |
| EEPGLPPQ**PQPPPPPLPQ**-PGDKSSWG | *Hоmо sарiеns* |
| EEPGLPPQ**PQPQPPPLPQ**-PGDKSSWG | *Раn trоglоdуtеs* |
| EEPGLPPQ**PQPQPPPLPQ**-PGDKSSWG | *Раn раnisсиs* |
| EEPGLPPQ**PQPQPQSPLPQ**PGDKNSWG | *Рарiо Аnubis* |
| EEPGLPPQ**PQPQPQSPLPQ**PGDKNSWG | *Mасаса mulаttа* |
| DEPPTSPR**QLQPAPPLAPQ**-GRPWTPG | *Gоrillа gоrillа* |
| Oligopeptides encoded by miRNA binding sites are in bold. |  |
