# Supplementary material for: In silico Prediction of miRNA Interactions With Candidate Atherosclerosis Gene mRNAs
Source: Front Genet. 2020 Nov 4;11:605054. doi: 10.3389/fgene.2020.605054 (PMC7672156; doi:10.3389/fgene.2020.605054)
Supplement: Supplementary file 4 [file Table_4.DOCX]

**Table S4.** Characteristics of miRNAs interaction in the CDS of mRNA of atherosclerosis candidate genes

| **Gene** | **miRNA** | **Start of site, nt** | **∆G, kJ/mole** | **∆G/∆Gm, %** | **Length, nt** |
| --- | --- | --- | --- | --- | --- |
| *ACE* | ID00522.5p-miR | 62 | -125 | 89 | 23 |
|  | ID02294.5p-miR | 64 | -132 | 90 | 24 |
|  | ID01879.5p-miR | 94 | -123 | 91 | 22 |
| *ADAMTS13* | ID01721.3p-miR | 2718 | -121 | 89 | 24 |
|  | ID00196.3p-miR | 3585 | -121 | 90 | 23 |
| *ADAMTS7* | ID01310.3p-miR | 877 | -121 | 92 | 22 |
|  | ID03030.3p-miR | 5030 | -125 | 89 | 24 |
| *ADRB3* | ID01901.5p-miR | 1306 | -121 | 92 | 21 |
|  | ID00009.3p-miR | 1312 | -115 | 92 | 20 |
|  | ID02770.5p-miR | 1312 | -115 | 92 | 20 |
|  | ID02781.3p-miR | 1313 | -115 | 92 | 20 |
|  | ID02036.3p-miR | 1314 | -115 | 92 | 20 |
| *ANGPTL4* | ID01593.5p-miR | 259 | -134 | 100 | 23 |
| *APOB* | ID00843.5p-miR | 179 | -119 | 90 | 23 |
| *APOE* | ID03402.5p-miR | 643 | -121 | 95 | 22 |
| *CDKN1C* | miR-762 | 738÷744 (2) | -123÷-125 | 91÷92 | 22 |
|  | ID03129.3p-miR | 753 | -119 | 97 | 20 |
|  | miR-762 | 804÷900 (17) | -123÷-132 | 91÷97 | 22 |
|  | ID00099.3p-miR | 824÷836 (3) | -113 | 93 | 21 |
|  | ID02682.5p-miR | 831 | -113 | 93 | 20 |
|  | ID00036.3p-miR | 888 | -121 | 90 | 22 |
|  | ID01075.3p-miR | 894 | -125 | 89 | 23 |
|  | ID00411.5p-miR | 895 | -129 | 90 | 23 |
| *FASLG* | ID00061.3p-miR | 290÷326(4) | -125÷-127 | 91÷92 | 22 |
|  | ID00296.3p-miR | 314÷318(3) | -138 | 88 | 25 |
|  | ID01641.3p-miR | 318 | -134 | 90 | 24 |
|  | ID01702.3p-miR | 321 | -134 | 89 | 24 |
| *IRS2* | ID00776.3p-miR | 518 | -123 | 94 | 22 |
|  | ID02541.5p-miR | 944 | -121 | 90 | 22 |
|  | ID00767.3p-miR | 972 | -127 | 91 | 23 |
|  | ID01702.5p-miR | 975 | -138 | 88 | 25 |
|  | ID02260.5p-miR | 975 | -127 | 91 | 22 |
|  | ID01560 .3p-miR | 1614 | -127 | 92 | 23 |
|  | ID00872.3p-miR | 1627 | -121 | 89 | 23 |
|  | ID01336.3p-miR | 1627 | -134 | 87 | 24 |
|  | ID03332.3p-miR | 1630 | -132 | 89 | 24 |
|  | ID01190.5p-miR | 1825 | -138 | 90 | 24 |
|  | ID01190.5p-miR | 3293 | -136 | 89 | 24 |
|  | miR-6806-5p | 4444 | -123 | 91 | 24 |
| *KLF2* | ID00457.3p-miR | 576 | -123 | 91 | 22 |
|  | ID01458.5p-miR | 571 | -134 | 91 | 23 |
| *MMP2* | ID02146.5p-miR | 379 | -125 | 91 | 23 |
| *NOS3* | ID01035.3p-miR | 2946 | -123 | 89 | 24 |
| *PDE4D* | miR-3960 | 336 | -117 | 93 | 20 |
|  | ID01155.3p-miR | 344 | -129 | 94 | 22 |
|  | ID00061.3p-miR | 344 | -129 | 94 | 22 |
|  | ID01702.3p-miR | 338÷345(3) | -134 | 89 | 24 |
|  | ID03064.3p-miR | 344 | -136 | 89 | 24 |
|  | ID00061.3p-miR | 391÷413(3) | -125÷-129 | 91÷94 | 22 |
|  | ID00296.3p-miR | 404÷411(4) | -140÷-142 | 89÷91 | 25 |
|  | ID00457.3p-miR | 392 | -123 | 91 | 22 |
|  | ID01155.3p-miR | 391÷395(2) | -125÷-129 | 91÷94 | 22 |
|  | ID01184.3p-miR | 419 | -117 | 93 | 20 |
|  | ID01315.3p-miR | 392 | -115 | 92 | 20 |
|  | ID01377.3p-miR | 394 | -121 | 95 | 20 |
|  | ID01458.5p-miR | 410 | -134 | 91 | 23 |
|  | ID01641.3p-miR | 407÷410(2) | -132÷-132 | 89 | 24 |
|  | ID01702.3p-miR | 411 | -138 | 92 | 24 |
|  | ID01705.3p-miR | 398 | -117 | 92 | 21 |
|  | ID02064.5p-miR | 415 | -129 | 90 | 23 |
|  | miR-3960 | 408÷415(3) | -115÷-117 | 92÷93 | 20 |
| *PLTP* | ID00573.5p-miR | 1763 | -121 | 90 | 23 |
| *RTN4* | ID01653.5p-miR | 502 | -134 | 90 | 24 |
|  | miR-6894-5p | 714 | -121 | 90 | 24 |
| *SCAP* | ID00792.3p-miR | 2486 | -125 | 91 | 22 |
| *SIRT1* | miR-4767 | 235 | -134 | 94 | 23 |
|  | ID01560.3p-miR | 264 | -123 | 89 | 23 |
|  | ID03332.3p-miR | 280÷287(2) | -132÷-138 | 89÷93 | 24 |
|  | ID00278.3p-miR | 294 | -123 | 89 | 23 |
|  | ID00811.3p-miR | 300 | -125 | 88 | 24 |
| *TBC1D10B* | miR-762 | 228 | -136 | 100 | 22 |
| *THBD* | ID02538.3p-miR | 709 | -121 | 90 | 22 |
| *VEGFA* | ID03097.3p-miR | 888 | -121 | 89 | 23 |
| *VWF* | ID01238.5p-miR | 5029 | -127 | 90 | 24 |
| *UTS2R* | ID03390.3p-miR | 860 | -127 | 88 | 24 |
