# Supplementary material for: In silico Prediction of miRNA Interactions With Candidate Atherosclerosis Gene mRNAs
Source: Front Genet. 2020 Nov 4;11:605054. doi: 10.3389/fgene.2020.605054 (PMC7672156; doi:10.3389/fgene.2020.605054)
Supplement: Supplementary file 5 [file Table_5.DOCX]

**Table S5.** Oligopeptides encoded by miR-762 binding sites located in mRNA coding region of orthologous gene *TBC1D10B.*

| Plots of proteins containing oligopeptides encoded by mRNA binding sites of orthologous gene *TBC1D10B* | Object |
| --- | --- |
| AWVPGSAQTS**APAPAPAPAPA**....VTGSTVVVLTL | *Рарiо Аnubis* |
| AWVPGSAQTS**APAPAPAPAPA**....VTGSTVVVLTL | *Macaca mulatta* |
| AWVPGSAQTS**APAPAPAPA**......VTGSTVVVLTL | *Gоrillа gоrillа* |
| AWVPGSAQTS**APAPAPAPA**......VTGSTVVVLTL | *Nоmаsсus lеuсоgеnуs* |
| AWVPGSAQTS**APAPAPAPA**......VTGSTVVVLTL | *Сhlоrосеbus sаbаеus* |
| AWVPGSAQTS**APAPAPAPA**......VTGSTVVVLTL | *Mасаса fаsсiсulаris* |
