# Supplementary material for: In silico Prediction of miRNA Interactions With Candidate Atherosclerosis Gene mRNAs
Source: Front Genet. 2020 Nov 4;11:605054. doi: 10.3389/fgene.2020.605054 (PMC7672156; doi:10.3389/fgene.2020.605054)
Supplement: Supplementary file 6 [file Table_6.DOCX]

**Table S6.** Oligopeptides encoded by miR-762 binding sites located in mRNA coding region of orthologous gene *CDKN1C.*

| Plots of proteins containing oligopeptides encoded by mRNA binding sites of orthologous gene *CDKN1C* | Object |
| --- | --- |
| AAPVAVAVLA**PAPVLAPVPVPAPAPVVAPAPAPAPVLAPAPVLAPAPAPVVAPAPAPAPVVAPAPAPAPAPAPAPAQ**DAAPQESAEQ | *Сhlоrосеbus sаbаеus* |
| AAPVAVAVLA**PAPVLAPAPAPAPAPAPAPAPAPAPVVAPAPAPAPVVAPAPAPAPAPAPVPAPAPAPAQ**--------DAAPQESAEQ | *Macaca mulatta* |
| AAPVAVAVLA**PAPAPAPVPVPAPAPAPAPAPAPAPAPVVAPAPAQAPAPAPAPAPAPAPAPAPAQ**------------DAAPQESAEQ | *Rhinорithесus rоxеllаnа* |
| AAPVAVAVLA**PAPVPAPAPAPAPAPAPAPAPAPVAAPAPAPAPAPAPAPAPAPAPAPAP**------------------DAAPQESAEQ | *Роngо аbеlii* |
| AAPVAVAVLA**PAPAPAPAPAPAPVAAPAPAPAPAPAPAPAPAPAPAP**------------------------------DAAPQESAEQ | *Nоmаsсus lеuсоgеnуs* |
| AAPVAVAVLA**PAPAPAPAPAPAPAPVAAPAPAPAPAPAPAPAPAPAP**------------------------------DAAPQESAEQ | *Hоmо sарiеns* |
| AAPVAVAVLA**PAPAPAPAPAPAPVAAPAPAPAPAPAPAPAPAPAP**--------------------------------DAAPQESAEQ | *Раn trоglоdуtеs* |
