# Supplementary material for: In silico Prediction of miRNA Interactions With Candidate Atherosclerosis Gene mRNAs
Source: Front Genet. 2020 Nov 4;11:605054. doi: 10.3389/fgene.2020.605054 (PMC7672156; doi:10.3389/fgene.2020.605054)
Supplement: Supplementary file 7 [file Table_7.DOCX]

**Table S7.** Characteristics of miRNAs interaction in the 3'UTR of mRNA of atherosclerosis candidate genes

| **Gene** | **miRNA** | **Start of**  **site, nt** | **ΔG, kJ/mole** | **ΔG/ΔGm,**  **%** | **Length,**  **nt** |
| --- | --- | --- | --- | --- | --- |
| *ABO* | ID01694.5p-miR | 1094 | -117 | 92 | 22 |
|  | ID01382.3p-miR | 1125 | -113 | 93 | 20 |
| *ACE* | ID00850.3p-miR | 4068 | -117 | 90 | 22 |
|  | ID00092.3p-miR | 4657 | -110 | 90 | 22 |
| *ADAM17* | ID02997.5p-miR | 3449 | -113 | 93 | 22 |
|  | miR-619-5p | 3465 | -121 | 100 | 22 |
|  | miR-1285-5p | 3523 | -104 | 92 | 21 |
| *ADAM33* | miR-619-5p | 3227 | -117 | 96 | 22 |
|  | miR-1303 | 3477 | -113 | 96 | 22 |
| *ANGPT2* | ID03006.5p-miR | 3064 | -121 | 89 | 24 |
|  | miR-7110-3p | 4996 | -110 | 91 | 22 |
| *APOL1* | miR-619-5p | 2122 | -115 | 95 | 22 |
|  | ID01237.3p-miR | 2363 | -115 | 90 | 24 |
| *BRCA1* | miR-5095 | 6405 | -106 | 91 | 21 |
|  | miR-619-5p | 6411 | -119 | 98 | 22 |
|  | miR-5096 | 6485 | -110 | 98 | 21 |
|  | miR-5585-3p | 6553 | -110 | 95 | 22 |
| *CD59* | ID00101.3p-miR | 6690 | -117 | 93 | 22 |
| *GAS6* | miR-1914-5p | 2032 | -115 | 90 | 22 |
|  | miR-1914-5p | 2365 | -115 | 90 | 22 |
|  | ID03062.3p-miR | 2373 | -119 | 90 | 22 |
| *F11R* | ID01640.5p-miR | 1909 | -117 | 89 | 24 |
|  | miR-5096 | 1946 | -108 | 96 | 21 |
|  | miR-619-5p | 2000 | -113 | 93 | 22 |
|  | miR-5585-3p | 2007 | -106 | 91 | 22 |
|  | ID00367.5p-miR | 3291 | -110 | 90 | 22 |
|  | miR-1273g-3p | 3297 | -115 | 98 | 21 |
|  | miR-1972 | 3536 | -110 | 90 | 22 |
| *FADS2* | ID01604.3p-miR | 2407 | -117 | 89 | 23 |
|  | ID00204.5p-miR | 2712 | -117 | 90 | 22 |
|  | miR-1224-3p | 2762 | -115 | 96 | 21 |
|  | ID01330.3p-miR | 2803 | -119 | 89 | 23 |
| *FOXP3* | miR-762 | 1691 | -125 | 92 | 22 |
|  | miR-5088-5p | 2252 | -123 | 92 | 24 |
| *GPR132* | miR-1273d | 3312 | -123 | 91 | 25 |
| *HNF1A* | miR-3605-5p | 2564 | -110 | 90 | 23 |
| *IGF1* | miR-1273g-3p | 6008 | -113 | 96 | 21 |
|  | miR-1273f | 6041 | -102 | 98 | 19 |
|  | miR-1273e | 6051 | -108 | 93 | 22 |
| *IGF1R* | ID01901.5p-miR | 5113 | -121 | 92 | 21 |
|  | ID00928.3p-miR | 6030 | -113 | 91 | 23 |
| *ITGA2* | miR-5095 | 5924 | -106 | 91 | 21 |
|  | miR-619-5p | 5930 | -110 | 91 | 22 |
|  | miR-5096 | 6002 | -110 | 98 | 21 |
| *LDLR* | miR-5585-3p | 4042 | -113 | 96 | 22 |
|  | miR-619-5p | 4378 | -113 | 93 | 22 |
|  | miR-619-5p | 4516 | -113 | 93 | 22 |
|  | ID02566.3p-miR | 4560 | -110 | 90 | 22 |
|  | ID01836.5p-miR | 4608 | -115 | 92 | 23 |
| *LPCAT3* | miR-1273a | 1759 | -119 | 90 | 25 |
|  | ID00611.5p-miR | 1977 | -115 | 89 | 23 |
|  | miR-1972 | 2020 | -113 | 91 | 22 |
| *MTHFR* | ID03245.5p-miR | 3350 | -117 | 89 | 24 |
|  | ID00345.3p-miR | 6281 | -119 | 89 | 23 |
|  | ID01811.5p-miR | 6844 | -117 | 93 | 22 |
|  | miR-5095 | 6854 | -110 | 95 | 21 |
|  | miR-619-5p | 6860 | -115 | 95 | 22 |
|  | miR-5585-3p | 7002 | -110 | 95 | 22 |
|  | ID02175.3p-miR | 7051 | -113 | 93 | 22 |
| *NOS1AP* | ID02430.3p-miR | 3006 | -121 | 89 | 23 |
|  | ID01727.5p-miR | 4607÷4611(2) | -106 | 91 | 23 |
| *NR4A2* | ID02299.5p-miR | 2605÷2615(3) | -98 | 92 | 21 |
|  | ID00470.5p-miR | 2606 | -110 | 91 | 23 |
| *OLR1* | miR-574-5p | 1503÷1505(2) | -113 | 93 | 23 |
|  | ID00470.5p-miR | 1504÷1506(2) | -108 | 89 | 23 |
| *PNPLA3* | miR-619-5p | 2461 | -113 | 93 | 22 |
|  | miR-5585-3p | 2468 | -108 | 93 | 22 |
| *PPARA* | ID00913.5p-miR | 2293 | -115 | 90 | 23 |
|  | miR-1913 | 3949 | -115 | 90 | 22 |
| *SOAT1* | ID01404.5p-miR | 5523 | -110 | 90 | 23 |
|  | miR-619-5p | 5867 | -110 | 91 | 22 |
| *SOCS3* | miR-1237-3p | 1854 | -110 | 93 | 21 |
| *TFPI* | ID00367.5p-miR | 2697 | -110 | 90 | 22 |
|  | miR-1273g-3p | 2703 | -115 | 98 | 21 |
| *TNC* | miR-5585-3p | 7989 | -110 | 95 | 22 |
|  | ID01836.5p-miR | 8073 | -115 | 92 | 23 |
| *TNFSF10* | miR-619-5p | 1449 | -110 | 91 | 22 |
|  | miR-619-5p | 1582 | -115 | 95 | 22 |
|  | miR-5585-3p | 1589 | -108 | 93 | 22 |
|  | ID02175.3p-miR | 1638 | -113 | 93 | 22 |
| *ZBTB46* | ID00648.5p-miR | 2401 | -125 | 92 | 22 |
|  | ID00382.5p-miR | 3819 | -113 | 93 | 20 |
|  | ID02460.5p-miR | 4010 | -117 | 90 | 22 |
|  | ID03288.5p-miR | 4488 | -117 | 90 | 23 |
